# Red Cell Transfusion and Outcomes in Cancer Surgery—Another Piece of the Jigsaw

**DOI:** 10.1213/ANE.0000000000007480

**Published:** 2025-04-04

**Authors:** Akshay Shah, Sonali V. Thakrar, Christopher J. Peters, Sanooj Soni

**Affiliations:** 1 Department of Anaesthesia, Hammersmith Hospital, Imperial College Healthcare NHS Trust, London, UK, Nuffield Department of Clinical Neurosciences, University of Oxford, Oxford, UK, Akshay.shah@linacre.ox.ac.uk; 2 Department of Anaesthesia, Hammersmith Hospital, Imperial College Healthcare NHS Trust, London, UK; 3 Department of Surgery and Cancer, Imperial College London, London, UK; 4 Division of Anaesthetics, Pain Medicine, and Intensive Care, Department of Surgery & Cancer, Imperial College London London, UK

## To the Editor

We read with great interest the study on the association between perioperative red blood cell (RBC) transfusion at 1-year mortality after major cancer surgery by Cata et al.^1^ The authors concluded that perioperative RBC transfusion is associated with an increased cancer risk. However, these findings should be interpreted with caution for the following reasons.

Previous meta-analyses, pooling data from observational studies, have mostly been of low to critically low quality, with minimal control for residual confounding.^2^ Although Cata et al attempted to address this by including some important prognostic covariates (eg, preoperative anemia) in their multivariate logistic model, several other potentially important ones were omitted. These include frailty, cardiovascular comorbidities, socioeconomic status, preoperative neoadjuvant chemo/radiotherapy, surgical approach (open versus minimally invasive), estimated blood loss, surgical urgency and use of cointerventions (eg, tranexamic acid, cell salvage). It is hardly surprising that Cata et al^1^ found that patients who experienced more intraoperative blood loss and those who underwent more complex surgery (eg, open, multi-organ, longer duration) were more likely to receive an RBC transfusion, but equally this group may also inherently be at greater risk of cancer recurrence due to the nature of the more invasive surgery. Sicker patients, who are already at an increased baseline risk of poor outcomes, are also more likely to receive a transfusion.

The study also highlights an apparent dose-dependent effect of transfusion on mortality, with increasing transfusion volume associated with worse outcomes. However, this finding may reflect the severity of illness in transfused patients rather than a direct causal relationship. Indeed, the fact that after propensity score matching, the difference in 1-year mortality between transfused (17.4%) and nontransfused (13.2%) patients did not reach statistical significance suggests that residual confounding may still be present, and overgeneralization of these findings should be avoided.

Combining different types of cancer surgery into 1 analysis also poses challenges due to differences in perioperative transfusion thresholds. For example, in our institution, a strict restrictive transfusion threshold strategy in the absence of ischemic heart disease (transfuse if hemoglobin (Hb) <70 g.L^−1^) is adopted for patients undergoing esophageal cancer surgery. Conversely, in patients undergoing ovarian debulking surgery, a liberal transfusion threshold is recommended, and fresh frozen plasma for volume replacement, the latter particularly in patients with gross ascites undergoing multivisceral resections.^3^ Indeed, perioperative plasma transfusion, in addition to RBC transfusion, has been shown to be associated with worse clinical outcomes when compared with RBC transfusion.^4^

An important consideration for a primary outcome such as all-cause 1-year mortality is the attributable fraction, which is defined as the proportional reduction in mortality that would occur if exposure to a risk factor (ie, RBC transfusion in this instance) were reduced to an alternative ideal exposure (eg, no transfusion). In critically ill patients with sepsis, the attributable fraction of sepsis to mortality has been shown to range from 15% to up to 93%.^5^ Sample size estimations based on eligibility criteria specific to attributable fraction of death due to transfusion could help improve the sensitivity of future studies.

Cata et al raise an important point around the use of Hb or physiological triggers to guide transfusion. Various physiological triggers have been proposed including hypotension, tachycardia, acidosis, ECG changes, ScvO_2_, lactate, and cerebral oximetry. However, we would like to add that in contrast to the large body of evidence evaluating Hb or hematocrit-based transfusions, there are few high-quality data evaluating the use of physiological transfusion triggers. Moreover, the variability in transfusion practices observed in the study, including inconsistent use of leukoreduced blood, further complicates interpretation.The relationship between RBC transfusion and adverse outcomes after cancer surgery remains complex and the authors should be commended on trying to untangle a piece of this jigsaw. Data science approaches with causal inference models and/or instrumental variable analysis, along with well-designed randomized trials are needed to provide high-quality data on the interplay between transfusion, different types of cancer, specific patient subgroups (frailty, cardiovascular disease), and meaningful patient-centered outcomes. Until then, reducing unnecessary transfusion should continue to be a focus of patient blood management in cancer surgery.^6^


**Akshay Shah, DPhil** *Department of Anaesthesia* *Hammersmith Hospital* *Imperial College Healthcare NHS Trust* *London, UK* *Nuffield Department of Clinical Neurosciences* *University of Oxford* *Oxford, UK* *Akshay.shah@linacre.ox.ac.uk*
**Sonali V. Thakrar, FRCA** *Department of Anaesthesia* *Hammersmith Hospital* *Imperial College Healthcare NHS Trust* *London, UK*
**Christopher J. Peters, PhD** *Department of Surgery and Cancer* *Imperial College London* *London, UK*
**Sanooj Soni, PhD** *Division of Anaesthetics, Pain Medicine, and Intensive Care* *Department of Surgery & Cancer* *Imperial College London London, UK*

